# Expression of connexin-43 in surgical resections of primary tumor and lymph node metastases of squamous cell carcinoma and adenocarcinoma of the lung: a retrospective study

**DOI:** 10.7717/peerj.13055

**Published:** 2022-03-09

**Authors:** Ivana Savic, Petar Milovanovic, Svetlana Opric, Nebojsa Ivanovic, Dejan Oprić

**Affiliations:** 1 Faculty of Medicine, University of Belgrade, Belgrade, Serbia; 2 University Hospital Bezanijska Kosa, Belgrade, Serbia

**Keywords:** Lung cancer, Connexin-43, Immunohistochemistry

## Abstract

**Background:**

Connexins are transmembrane proteins forming gap junctions between the cells, which allow intercellular communication. Significance of gap junctions and connexins in lung carcinoma is not yet understood. The objective of the study was to investigate immunohistochemical expression and the localization of connexin-43 (Cx43) in primary lung carcinoma and its lymphatic metastases.

**Methods:**

Surgical specimens of excised tumors from 88 patients (45 men and 43 women, 61.9 ± 7.4 years) with lung carcinoma (52 adenocarcinoma (AC), 36 squamous cell carcinoma (SqCC)) who were operated on at the University Hospital “Bezanijska Kosa” in a five-year period (2012–2016) were used. We conducted immunohistochemical staining for Cx43 and measured the degree of expression (percentage of positive cells and staining intensity) as well as localization of Cx43 in primary tumor and in lymphatic metastases.

**Results:**

Immunohistochemical analysis of the primary tumors revealed that SqCC showed significantly higher percentage of tumor cells expressing Cx43 as well as higher staining intensity than AC (*p* < 0.001). Almost 70% of samples with SqCC showed high Cx43 expression, whereas AC showed no expression in more than 50% of cases. Localization of Cx43 expression was most often cytoplasmic (AC and SqCC) and combined membranous and cytoplasmic (SqCC) with very rare instances of nuclear localization (AC). Almost the same pattern in distribution, intensity, and localization of Cx43 expression was observed in the lymph node metastases; however, almost a third of AC cases changed the pattern of Cx43 expression in the metastasis compared to primary tumor.

**Conclusion:**

The results of this study showed that lung carcinomas express Cx43 in more than 65% of cases and that it was aberrantly localized (not membranous localization). We highlighted that SqCC expressed Cx43 more than did AC, both in primary tumor and lymphatic metastases. Further research is needed to establish whether Cx43 could be used as a prognostic biomarker in lung carcinoma.

## Introduction

Lung cancer has been the leading cause of death among malignant tumors. According to World Health Organization, almost 1.7 million deaths worldwide each year occur due to lung cancer, which exceeds deaths from other types of cancer (Aasen, Sansano & Montero, 2019).

Lung carcinoma is classified into the following histological types: non-small cell lung carcinoma (encompassing squamous cell carcinoma (SqCC), adenocarcinoma (AC), and large cell carcinoma) and small-cell lung carcinoma. These histological types differ in biological behavior, consequently also in prognosis of the disease. Lung carcinoma spreads via lymphatic way to the regional lymph nodes, while most common hematogenous metastases are found in the liver, adrenal glands, brain, and bone marrow (Savic Milovanovic, Stjepanovic & Mitrovic, 2017).

Connexin-43 (Cx43) is a transmembrane protein forming intercellular *gap junctions* (Defamie, Chepied & Mesnil, 2014). These junctions allow rapid communication between the cells via direct exchange of small molecules, electrical signals, and ions (Defamie, Chepied & Mesnil, 2014). Cx43 is widely expressed molecule in many tissues, such as epithelial tissues, myocardium, neurons, liver, osteocytes (Defamie, Chepied & Mesnil, 2014; Plotkin & Bellido, 2013; Crespo Yanguas, Willebrords & Maes, 2016; Verheule & Kaese, 2013). Connexins are considered important for normal intercellular communication, as well as for normal growth and differentiation of tissues (Defamie, Chepied & Mesnil, 2014).

Considering that intercellular contact is an important element of carcinogenesis, it is of clear importance to evaluate the degree and localization of Cx43 expression in tumor cells of the primary tumor as well as metastatic deposits. However, the role of connexins in carcinogenesis is still insufficiently examined, and available data are conflicting. In previous studies on human material, researchers analyzed expression of Cx43 in ovarian cancer (Tanaka et al., 2016), breast carcinoma (Kanczuga-Koda, Sulkowski & Lenczewski, 2006), colonic carcinoma (Han et al., 2011), and melanoma (Tittarelli, Guerrero & Tempio, 2015). However, the data on lung cancers are particularly scarce (Zhao, Sun & Liu, 2013).

The purpose of this study was to analyze expression and localization of Cx43 as a marker of gap junctions in the tumor cells of the SqCC and AC of the lung, both in primary tumor and lymph node metastases.

## Materials & Methods

In this study we used surgical specimens of excised tumors from 88 patients (45 men, 43 women) with lung carcinoma who were operated on at the University Hospital “Bezanijska Kosa”, Belgrade in a 5-year period (2012–2016). The age range of these patients was 43–79 years (mean age: 61.9 ± 7.4 years).

Inclusion criteria encompassed: diagnosis of SqCC or AC of the lung, performed surgical resection of the tumor, and availability of the tissue blocks. Exclusion criteria encompassed: other types of lung carcinoma (including mixed types), lack of sufficient amount of tissue for immunohistochemical analyses, and presence of other malignant diseases.

Histological type of lung cancer was diagnosed during pathohistological evaluation of hematoxylin/eosin stained tissue sections along with standard immunohistochemical profiling when necessary.

AC was present in 52 patients and 36 patients had SqCC. There were no differences in age between these two groups of patients (*t* test: *p* = 0.8).

The study was approved by the institutional review board (Ethical Committee of the University Hospital “Bezanijska Kosa”, approval number: 6062/3).

### Imunohistochemical analysis

For immunohistochemical staining paraffin tissue blocks were cut to 4 µm sections. After deparaffinization, dehydratation, antigen retrieval, blocking of endogenous peroxidase and non-specific binding, the slides were treated with the primary antibody (rabbit anticonnexin-43 antibody; Sigma Aldrich) in 1:1200 concentration in 1% bovine serum albumin (BSA) in PBS overnight at 4 °C. Negative controls were treated in the same way except that primary antibody was omitted and 1% BSA in PBS was used instead. Next day, the slides were washed with PBS, and treated with polyclonal goat antirabbit Ig biotinylated (DAKO) dissolved in 1% BSA in PBS in ratio 1:200. After washing in PBS, streptavidin-HRP (1:200 in 1% BSA in PBS) was applied for 30 min. After staining with DAB, to facilitate visualization of tissue architecture and cells the slides were briefly soaked in hematoxylin.

Evaluation of Cx43 expression was performed in 10 tumor fields at 200x magnification in two ways: (1) by analyzing the percentage of tumor cells expressing Cx43 (distribution of expression) and (2) by analyzing Cx43 staining intensity in positive tumor cells.

In estimating the distribution of Cx43 expression, the percentage of tumor cells expressing Cx43 was classified as follows (Kanczuga-Koda, Sulkowski & Lenczewski, 2006):

• negative (percentage of stained tumor cells below 10%),
• intermediate expression (percentage of stained tumor cells between 10% and 50%), and
• high expression (percentage of stained tumor cells 50% or more).

The staining intensity was classified into four groups:

• intensity grade 0 (negative),
• intensity grade 1 (low intensity),
• intensity grade 2 (moderate intensity),
• intensity grade 3 (high intensity of staining).

We also noted the localization of immunohistochemical expression of Cx43 in the cells as membranous, cytoplasmic, combined membranous and cytoplasmic, nuclear, and combined nuclear and cytoplasmic.

Spread through air spaces (STAS) was also evaluated in all tumors, and expression of Cx43 was also marked in STAS.

### Statistical analysis

To compare the distribution, intensity, and localization between histological types we used Fisher’s exact probability test or chi-square test. *P*-values lower than 0.05 were considered statistically significant. SPSS software version 15 was used for all the analyses.

## Results

### Connexin-43 expression in primary tumor

**Figure 1:**
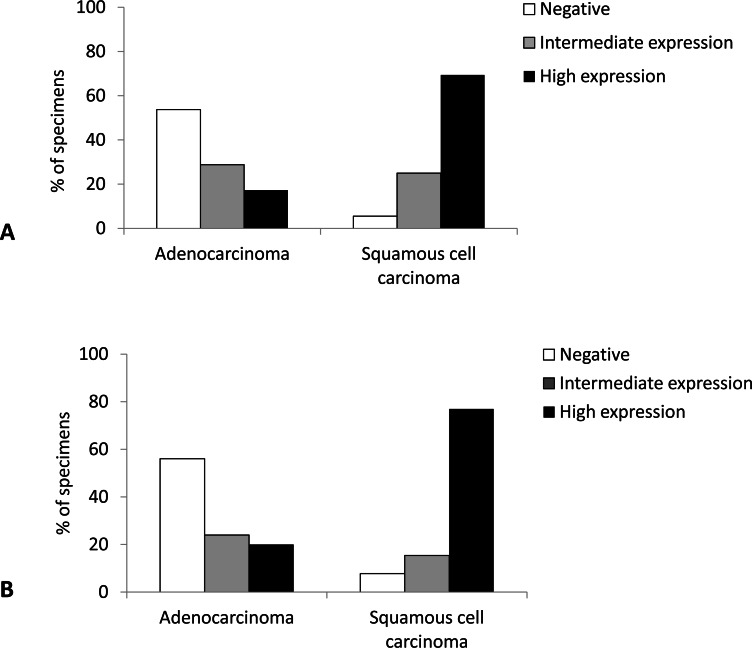
Distribution of expression of Cx43 in primary lung carcinoma (A) and lymph node metastasis (B). Negative, <10% of tumor cells stain; intermediate expression, 10%–50% of tumor cells stain; high expression, >50% of tumor cells stain.

Immunohistochemical analysis revealed that SqCC showed significantly higher Cx43 expression than AC (*p* < 0.001). Namely, almost 70% of samples with SqCC showed high Cx43 expression (more than 50% of tumor cells expressing Cx43), whereas AC showed no expression (<10% of positive cells) in more than 50% of cases (Fig. 1).

**Figure 2:**
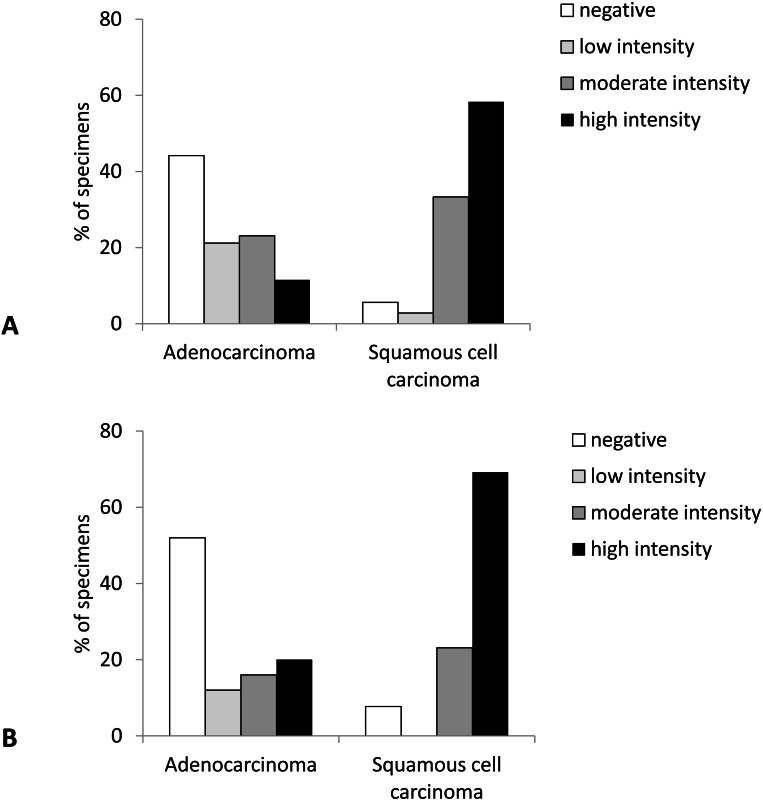
Intensity of Cx43 expression in primary lung carcinoma (A) and lymph node metastasis (B).

**Figure 3:**
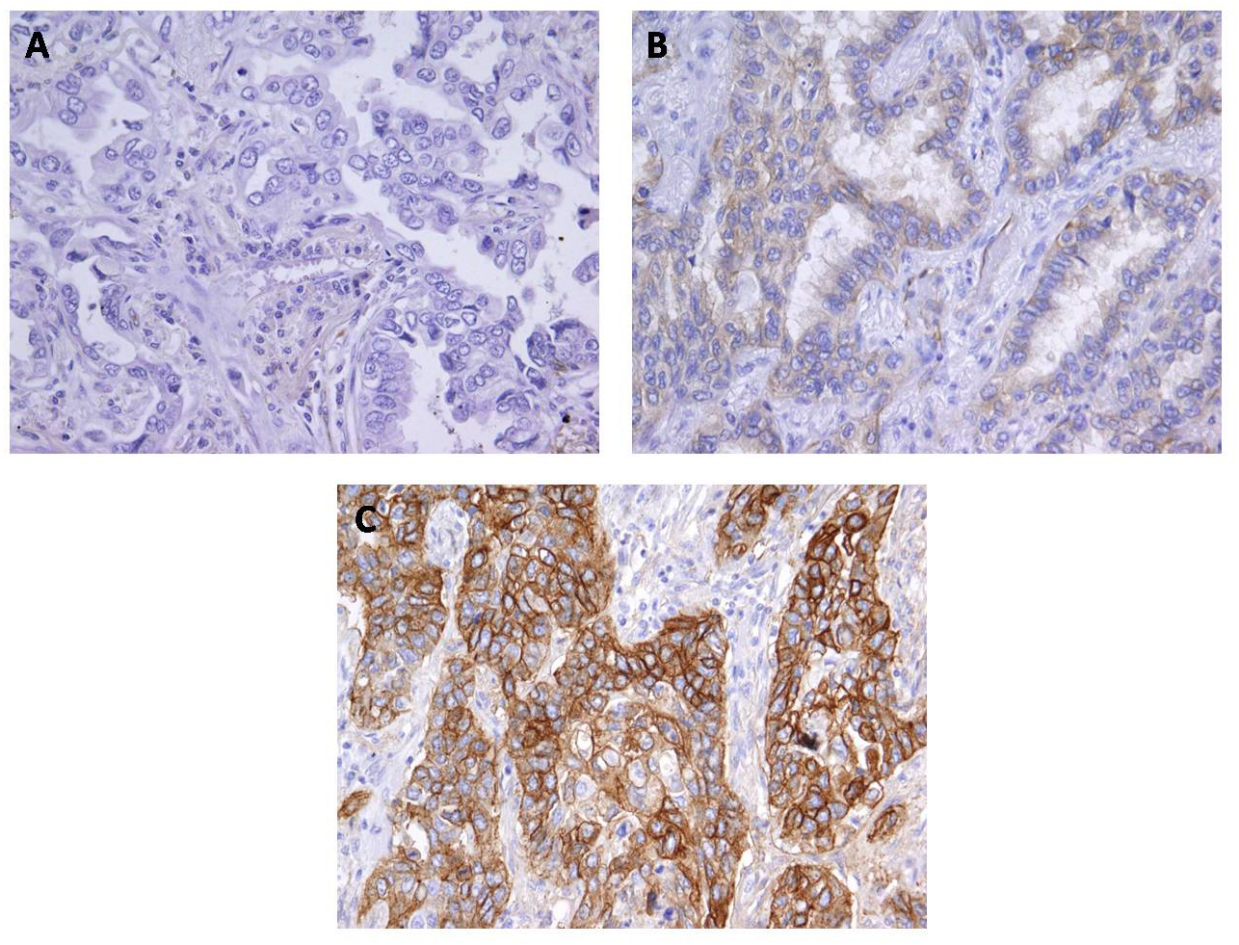
Immunohistochemical staining for Cx43 showing no expression (A) and low expression (B) in lung adenocarcinoma, and (C) high expression in squamous cell lung carcinoma. Magnification, ×400.

Likewise, the intensity of Cx43 expression was significantly higher in SqCC than in AC (*p* < 0.001). More than 90% of samples with SqCC showed high intensity of Cx43 expression (intensity grades 2 and 3) compared to around 30% of samples with AC (Figs. 2 and 3).

Among the analyzed 88 specimens, STAS was possible to assess in 68 specimens. Among them, STAS was observed in 11 out of 25 SqCC samples and 18 out of 43 AC samples (*p* = 0.341). The presence of STAS itself did not differ between those with vs. those without lymphatic metastases (*p* = 0.697 for SqCC, *p* = 1 for AC). However, Cx43 positivity in STAS tended to be higher in those with lymphatic metastases than in those without lymphatic metastases in SqCC (*p* = 0.055), but not in AC (*p* = 0.668).

**Figure 4:**
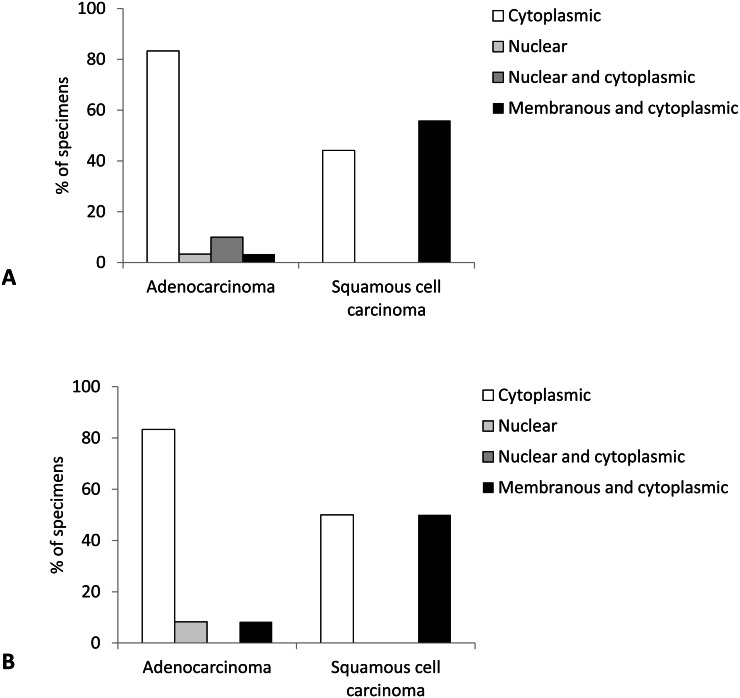
Localization of Cx43 expression in primary lung carcinoma (A) and lymph node metastasis (B).

**Figure 5:**
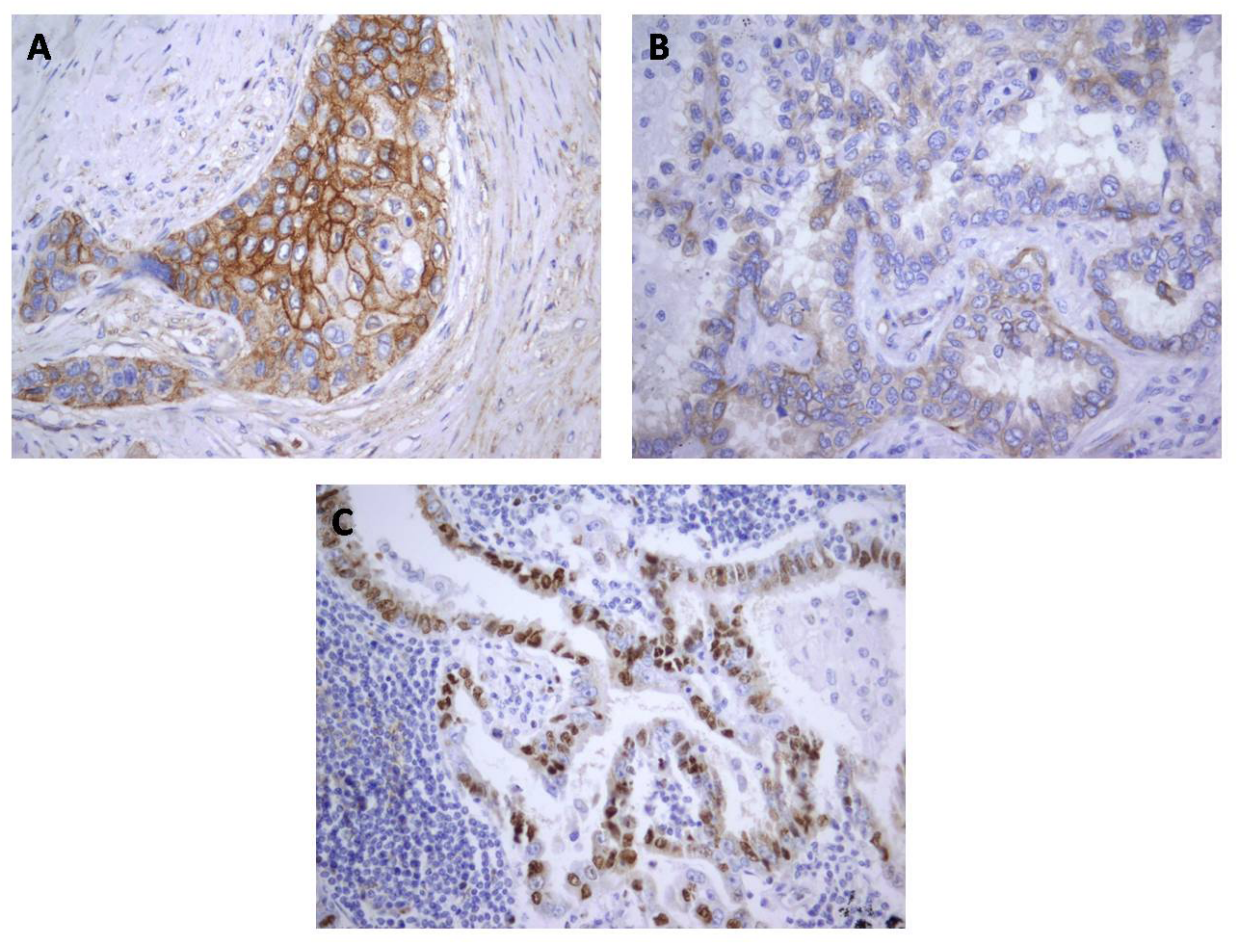
Localization of Cx43 expression in tumor cells: (A) Combined membranous and cytoplasmic; (B) Cytoplasmic; (C) Nuclear. Magnification, ×400.

Localization of Cx43 expression was most often cytoplasmic and combined membranous and cytoplasmic, with very rare instances of nuclear or combined nuclear and cytoplasmic localization. Comparison of tumor types revealed significant differences between AC and SqCC (*p* < 0.001). Namely, in AC Cx43 expression mostly occurred in cytoplasm (83.3% of cases), followed by rare combined nuclear and cytoplasmic, nuclear, and combined membranous and cytoplasmic localizations. In contrast, SqCC did not show any nuclear expression of Cx43, localizing exclusively to cytoplasm or combined membrane and cytoplasm of tumor cells, almost in similar amounts (Figs. 4 and 5).

### Connexin-43 expression in lymphatic metastasis

In the subset of patients (in whom the tumor had spread to the regional lymph nodes, *n* = 38), we had available tissue blocks both from the primary tumor and from the lymph node metastasis. In the lymph nodes of these patients, SqCC showed significantly higher distribution of Cx43 expression than AC (*p* = 0.002). Namely, high expression level (Cx43 positive in more than 50% of tumor cells) was observed in 76.9% of SqCC cases compared with only 20% of AC cases (Fig. 1).

Similarly, the intensity of Cx43 expression was shifted to higher values in SqCC (more than 90% showing staining intensity of grade 2 and 3) and no or low expression in AC samples (64% samples with the intensity scores 0 and 1) (*p* = 0.004) (Fig. 2).

Localization of Cx43 expression was most often cytoplasmic and combined membranous and cytoplasmatic, with only one sample with nuclear expression. There was a tendency to a significant difference in localization of Cx43 expression between the types of lung cancer (*p* = 0.069), with more than 80% of AC showing cytoplasmic only expression compared with balanced distribution of cytoplasmic only and combined membranous and cytoplasmic localizations of Cx43 in SqCC samples (Fig. 4).

### Differences in expression between the primary tumor and lymphatic metastasis

Comparison of Cx43 expression between the primary tumor and lymphatic metastases revealed that in most cases with AC (17/25) there was no change in the distribution of expression, and a decrease (or loss of expression) or increase (or appearance of expression) was noted in 20% and 12% of cases, respectively. In SqCC samples there was never a decrease/loss in Cx43 expression in lymphatic metastasis, while in one sample there was an increase in percentage of tumor cells expressing Cx43.

In terms of intensity of expression, SqCC in all but one case had similar intensity of Cx43 staining (intensity grades 2 and 3) between the primary and secondary tumor, while AC showed a decrease in staining intensity in 6/25 and an increase in intensity in 3/25 patients in lymph node compared to the primary location.

## Discussion

Our findings revealed that more than 65% of non-small cell lung carcinoma samples expressed Cx43 in primary tumors. Among them, SqCC had significantly higher Cx43 expression (higher percentage of Cx43 positive tumor cells) than AC, as well as higher intensity of expression. This is in disagreement with the data from a smaller study in Chinese patients where AC had higher expression of Cx43 mRNA (Zhao, Sun & Liu, 2013). Overall, the data regarding the role of Cx43 in tumorigenesis and metastatic process are contradictory in the literature. Namely, some authors showed higher Cx43 expression as a sign of progression to tumor (Brockmeyer et al., 2014), and higher Cx43 expression in tumors was regarded as a sign of worse prognosis, such as in case of esophageal SCC (Tanaka et al., 2016) and SqCC of oral cavity (Brockmeyer et al., 2014). Higher Cx43 expression was also related to higher adhesion of tumor cells to the pulmonary endothelium, which was associated with metastasis development in case of breast cancer (Elzarrad et al., 2008). Moreover, Cx43 was more expressed at the invasive front of colonic adenocarcinoma, and it was suggested that Cx43 may contribute to carcinogenesis either via *gap junctions* or via other independent signaling pathways, such as Wnt/ *β*-catenin (Han et al., 2011). In contrast, an animal study has shown that Cx43+/− mice presented significantly higher susceptibility to lung cancer, implying a relationship between Cx43 deficiency and carcinogenesis (De Oliveira et al., 2014). Likewise, other authors pointed to the lower rate of the tumor growth in melanoma highly expressing Cx43 (Tittarelli, Guerrero & Tempio, 2015) as well as in lung cancer cell line (Zhang et al., 1998). Moreover, Zhao, Han & Zhang (2011) showed in lung cancer cell culture that transfection with Cx43 reduced invasiveness and metastatic capacity via inhibition of histone deacetylases, but without reduction in cell migration.

Cx43 is a transmembranous protein, and that is why normal tissues display membranous positivity for Cx43 in immunohistochemical staining (Cogliati, Maes & Pereira, 2016). In contrast, previous studies noticed predominance of aberrant, cytoplasmic expression of Cx43 in tumors (Cogliati, Maes & Pereira, 2016; Brockmeyer et al., 2014), which was understood as its reduced transport to the plasma membrane or its increased internalization and degradation (Rose, Mehta & Loewenstein, 1993). While some authors suggested that aberrant Cx43 expression could promote oncogenesis (Cogliati, Maes & Pereira, 2016), a study on oral SqCC suggested that cytoplasmic Cx43 functions only as a depot, and that only membrane translocation of Cx43, which develops gap junctions, has influence on intercellular communication (Brockmeyer et al., 2014). Furthermore, the study on oral SqCC highlighted high membranous expression of Cx43 as an independent prognostic factor for poor overall survival (Brockmeyer et al., 2014). There are no comprehensive data regarding the subcellular distribution of connexins in lung cancers, but the data from breast cancer patients indicated mostly cytoplasmic Cx43 localization in primary tumors, while lymphatic metastases showed additionally marked membranous positivity (Kanczuga-Koda, Sulkowski & Lenczewski, 2006). In our study, we found aberrant localization of Cx43 expression, considering that it was never purely membranous, but rather solely cytoplasmic, combined membranous and cytoplasmic, and in rare instances even nuclear. While it is still unclear what the meaning of aberrant Cx43 expression in cancers is, lung SqCC had similar rate of purely cytoplasmic and combined membranous and cytoplasmic Cx43 expression, whereas in AC cytoplasmic expression predominated. Nuclear expression was only noticed in AC, and never in SqCC. The differential expression of Cx43 may reflect the differences in biological behavior between the tumor types. Moreover, it may reflect the differences in promoter methylation (Chen et al., 2003). Moreover, additional studies with larger patient series are warranted to explore the reasons for differential localization of Cx43 expression between SqCC and AC in the lungs as well as to clarify potential prognostic relevance of these differences.

Although Kanczuga-Koda, Sulkowski & Lenczewski (2006) showed higher Cx43 expression in lymphatic metastases compared to the primary breast cancer as well as different localization of Cx43 expression (membranous in lymph nodes vs. cytoplasmic in primary tumor), we noted that for lung cancer lymphatic metastases showed similar expression to the primary tumor in most cases. Nevertheless, the degree of Cx43 expression could increase or decrease in about 30% of patients with AC, and further studies are needed to examine whether transcriptome in lymph node metastases of AC could deviate significantly from the primary tumor and essentially could be a marker of tumor cell heterogeneity. The phenomenon of tumor cell heterogeneity is nowadays considered responsible for postsurgical relapse, resistance to treatment, and worse outcomes of various tumors (Stanta & Bonin, 2018; Guo et al., 2019). It should be noted that almost all SqCC cases in our study showed similar degree of Cx43 expression in secondary and primary tumor, which reflects the fact that primary tumor already shows maximum expression level in most SqCC cases. It is interesting that no decline in expression and no change in localization of expression was noted in case of SqCC.

STAS has attracted much attention lately and was associated with prognosis and tumor behavior in general (Warth, 2017). Our findings suggested that while the presence of STAS itself did not distinguish between those with vs. those without lymphatic metastases, it seems that Cx43 positivity in STAS increased the risk of lymphatic metastasis in SqCC with marginal significance, but not in AC. However, these data are based on a low number of samples with STAS in our study; therefore, larger cohorts are recommended to shed more light on the link between Cx43, STAS, and tumor behavior.

## Conclusions

Overall, our results showed that SqCC and AC lung cancers express Cx43, that this expression is aberrant in terms of subcellular localization, and also that Cx43 expression differs between SqCC and AC. While lymphatic metastases in SqCC showed equivalent expression of Cx43 as the primary tumors, in AC almost one-third of patients showed changed profile of Cx43 expression between the primary and secondary tumor deposits. Further studies are warranted to explore whether Cx43 could play a role in diagnostic evaluation of NSCLCs or whether it could be considered as prognostic factor or a target of new therapeutic approaches.

## Supplemental Information

10.7717/peerj.13055/supp-1 Supplemental Information 1 Database including demographic information, tumor types, and connexin-43 expression presented in this study

10.7717/peerj.13055/supp-2 Supplemental Information 2 Codebook of numerical grading of categorical variables

## Additional Information and Declarations

### Data Availability

The following information was supplied regarding data availability: The data is available in the Supplementary File.
