# Supplementary material for: Expression of connexin-43 in surgical resections of primary tumor and lymph node metastases of squamous cell carcinoma and adenocarcinoma of the lung: a retrospective study
Source: PeerJ. 2022 Mar 9;10:e13055. doi: 10.7717/peerj.13055 (PMC8917803; doi:10.7717/peerj.13055)
Supplement: Supplemental Information 2 [file peerj-10-13055-s002.pdf]

**Code book for categorical variables from the Supplemental data table**

**Tumor type**

- AC (adenocarcinoma of the lung)
- SqCC (squamous cell carcinoma of the lung)

**Distribution**

In estimating the distribution of Cx43 expression, the percentage of tumor cells expressing Cx43 in the slides was classified as follows:

- 0 (negative [percentage of stained tumor cells below 10%]),
- 1 (intermediate expression [percentage of stained tumor cells between 10% and 50%]), and
- 2 (high expression [percentage of stained tumor cells 50% or more]).

**Intensity**

The staining intensity was classified into four groups:

- 0 (negative),
- 1 (low intensity),
- 2 (moderate intensity),
- 3 (high intensity of staining).
